# Supplementary figures and images for: SPOP promotes ubiquitination and degradation of MyD88 to suppress the innate immune response
Source: PLoS Pathog. 2020 May 4;16(5):e1008188. doi: 10.1371/journal.ppat.1008188 (PMC7224567; doi:10.1371/journal.ppat.1008188)

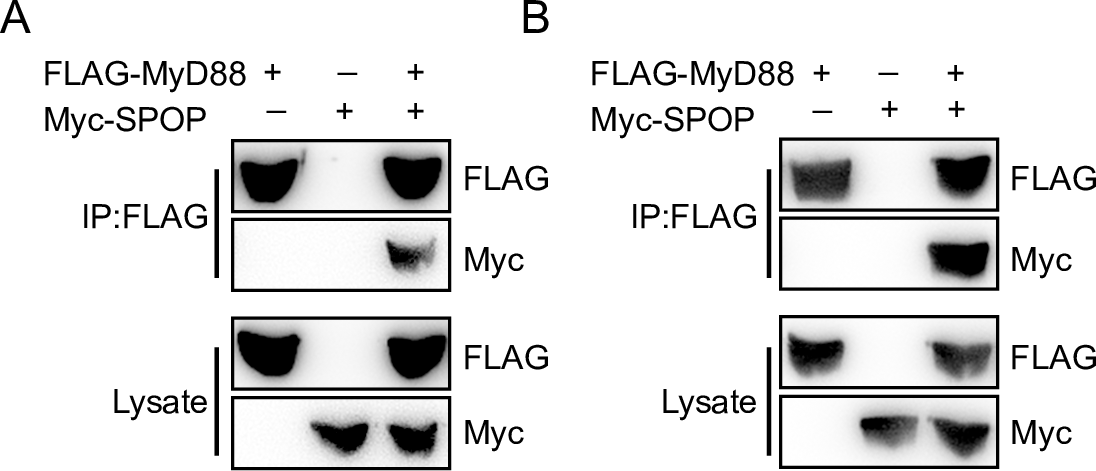

Supplement: S1 Fig — Hela cells and CHO cells were transfected with human (A) or mouse (B) MyD88 and SPOP. Immunoprecipitation using the anti-FLAG antibody was carried out to detect the interaction, followed by immunoblot analysis with indicated antibodies. (TIF) [file ppat.1008188.s001.tif]

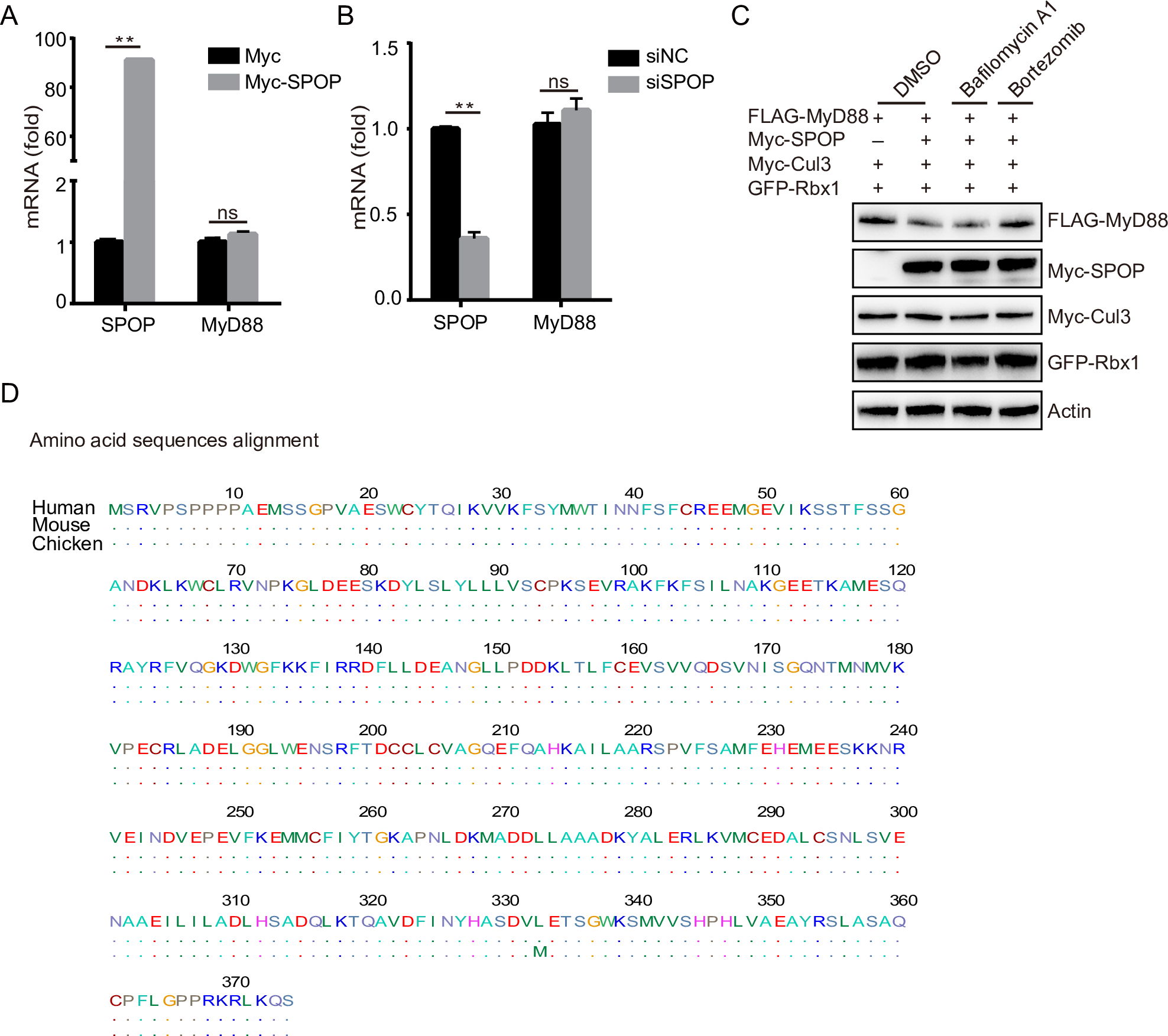

Supplement: S2 Fig — Expression of chSpop and chMyD88 mRNA in DF1 cells when chSPOP is (A) overexpressed or (B) knocked down. (C) Immunoblot analysis of chMyD88 in cell lysates of chicken DF1 cells transfected with chSPOP and treated with DMSO, 100 nM bafilomycin A, or 50 nM bortezomib. (D) Comparison of SPOP amino acid sequences in human, mouse, and chicken. (TIF) [file ppat.1008188.s002.tif]

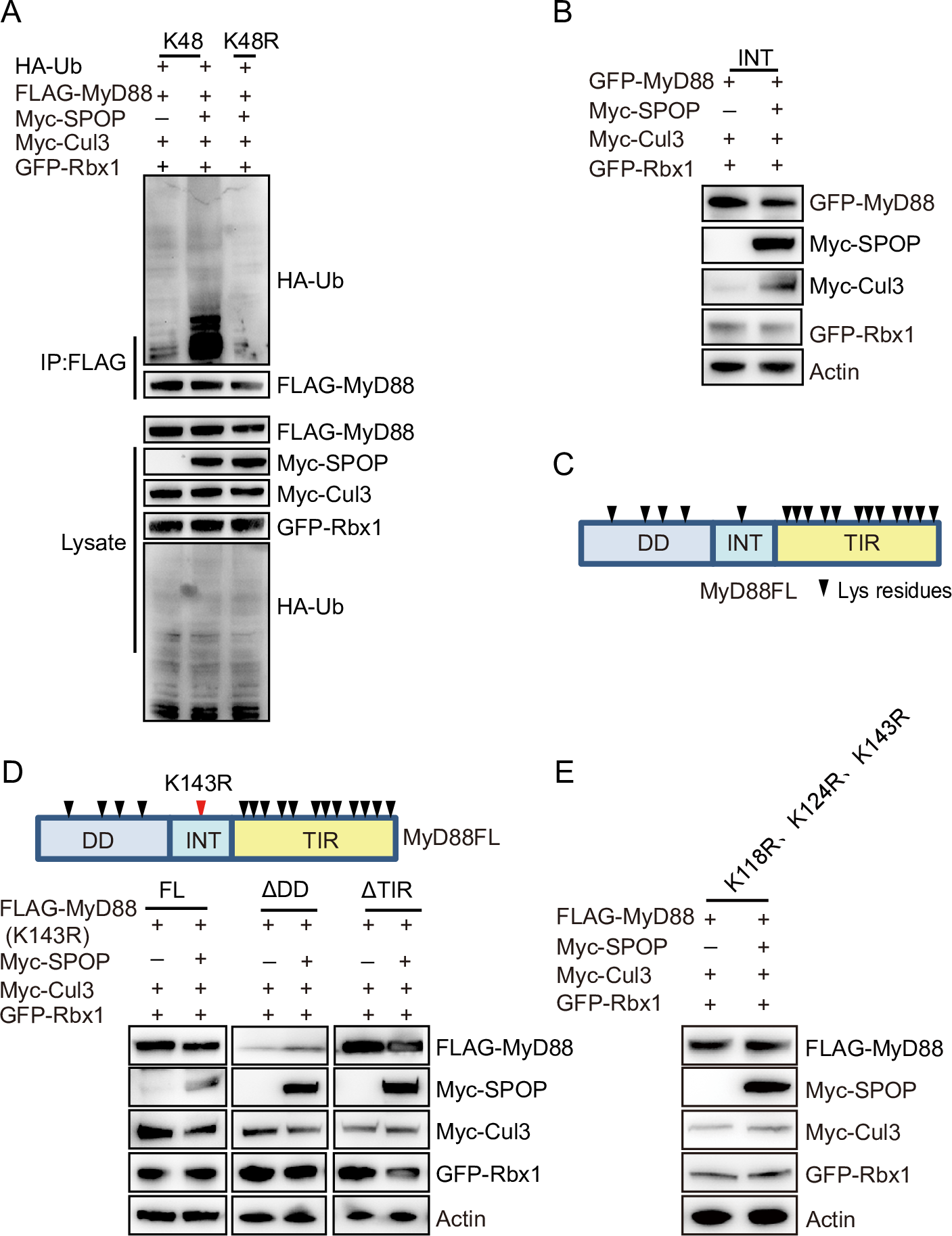

Supplement: S3 Fig — (A) Immunoblot analysis of immunoprecipitated chMyD88 from chicken DF1 cells transfected with indicated expression plasmids. (B) Immunoblot analysis of lysates from chicken DF1 cells transfected with GFP-tagged INT domain of chMyD88 and Myc-chSPOP. (C) Schematic diagram of the truncated chMyD88 mutants. (D) The DD domain of chMyD88 is required for the downregulation of chMyD88 by chSPOP. Indicated expression plasmids were co-transfected into chicken DF1 cells and the cell lysates immunoblotted with corresponding antibodies. (E) ChSPOP failed to downregulate K188, K124, and K143 triple mutated chMyD88 at the protein level. Immunoblot analysis of chMyD88 in cell lysates of chicken DF1 cells co-transfected with Myc-chSPOP. (TIF) [file ppat.1008188.s003.tif]

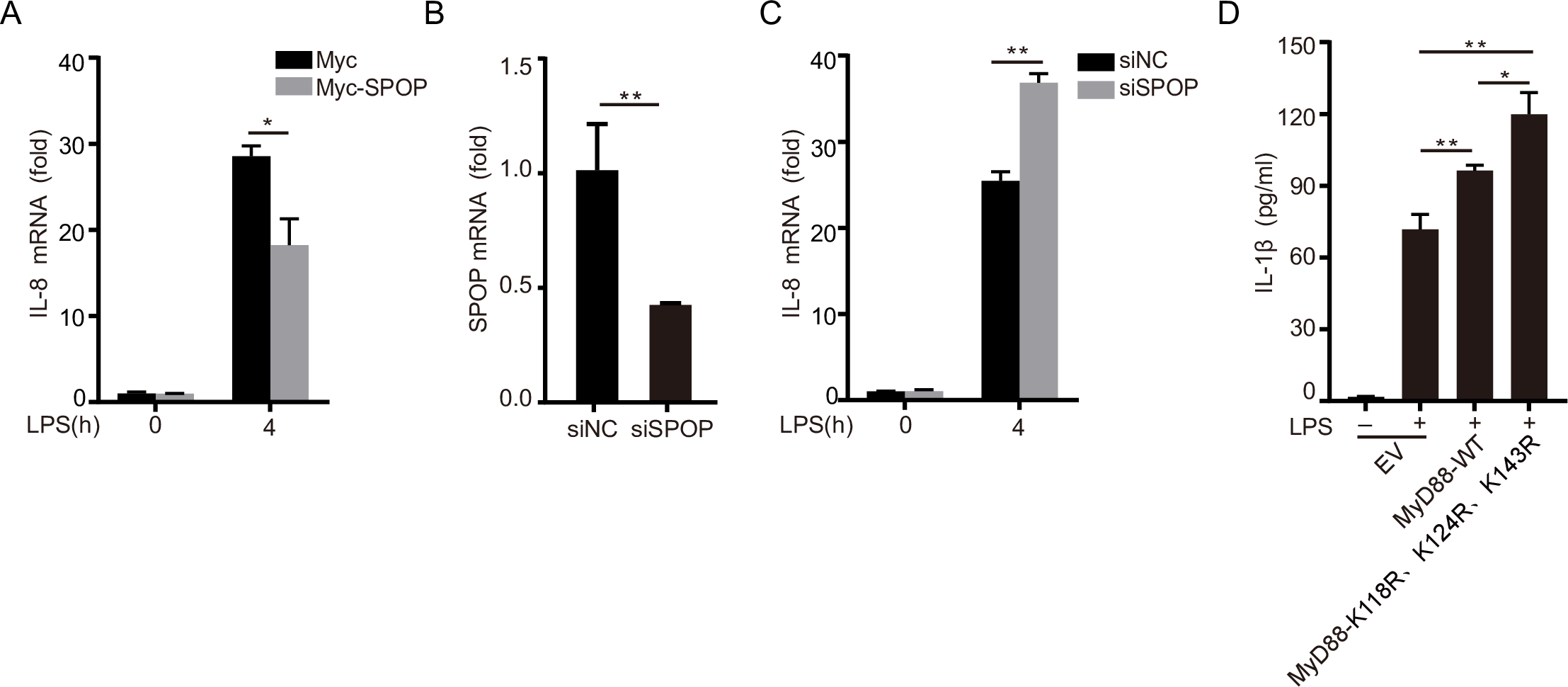

Supplement: S4 Fig — (A) Expression of IL-8 mRNA in chicken HD11 macrophages overexpressing chSPOP and stimulated with LPS for 4 h. (B) Real-time PCR analysis of chSPOP in chicken HD11 macrophage cells transfected with siRNA against chSPOP. (C) Expression of IL-8 mRNA in chicken HD11 macrophages transfected with siRNA against chSPOP and stimulated with LPS for 4 h. (D) ELISA of IL-1β in chicken HD11 macrophages transfected with triple mutant MyD88. *p < 0.05, **p < 0.01, error bars reflect ±SD. (TIF) [file ppat.1008188.s004.tif]

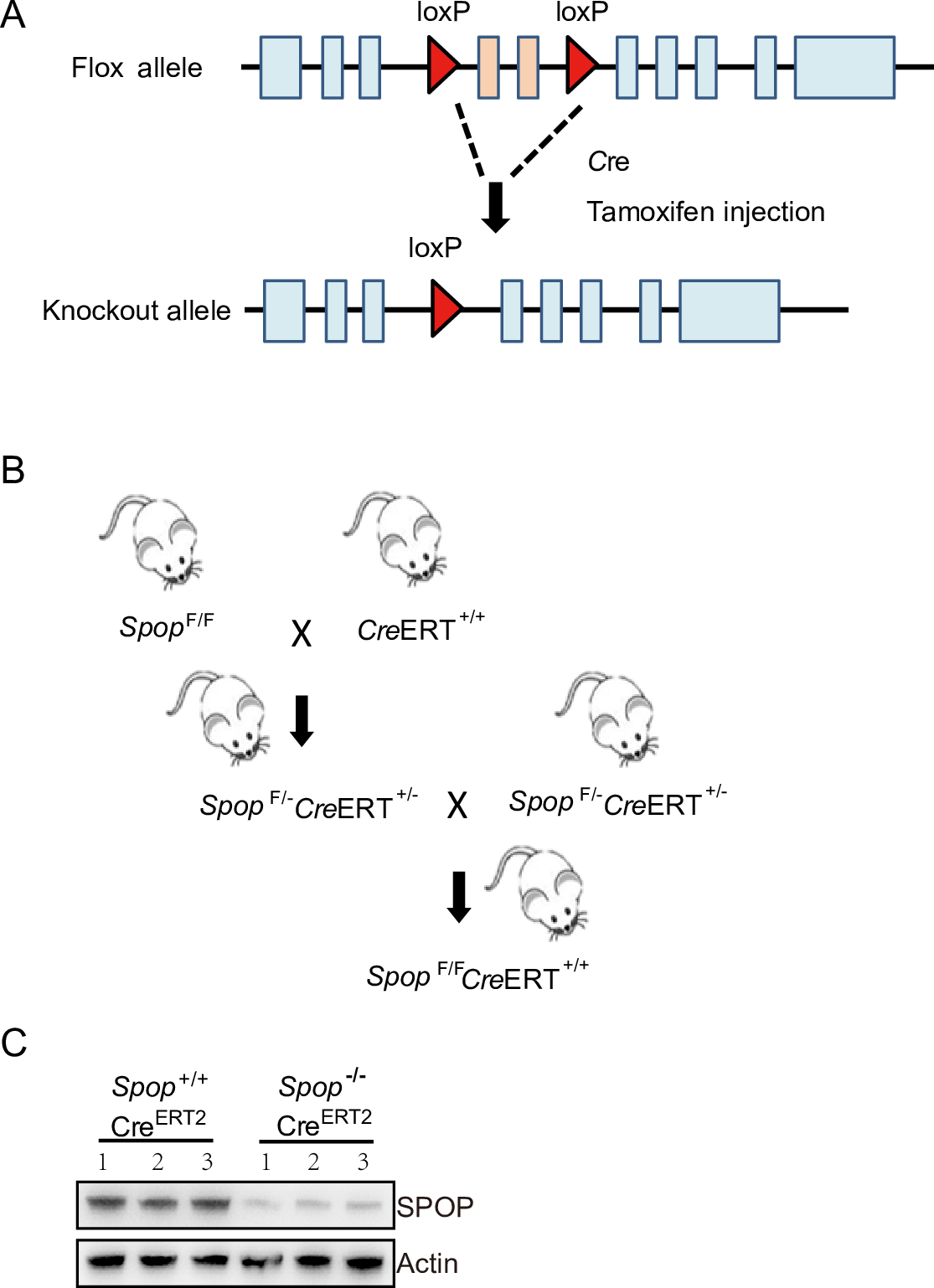

Supplement: S5 Fig — (A) and (B) Schematic diagram of Spop conditional knockout allele. (C) Immunoblot analysis of SPOP in the spleens of Spop+/+CreERT2 and Spop-/-CreERT2 mice. (TIF) [file ppat.1008188.s005.tif]

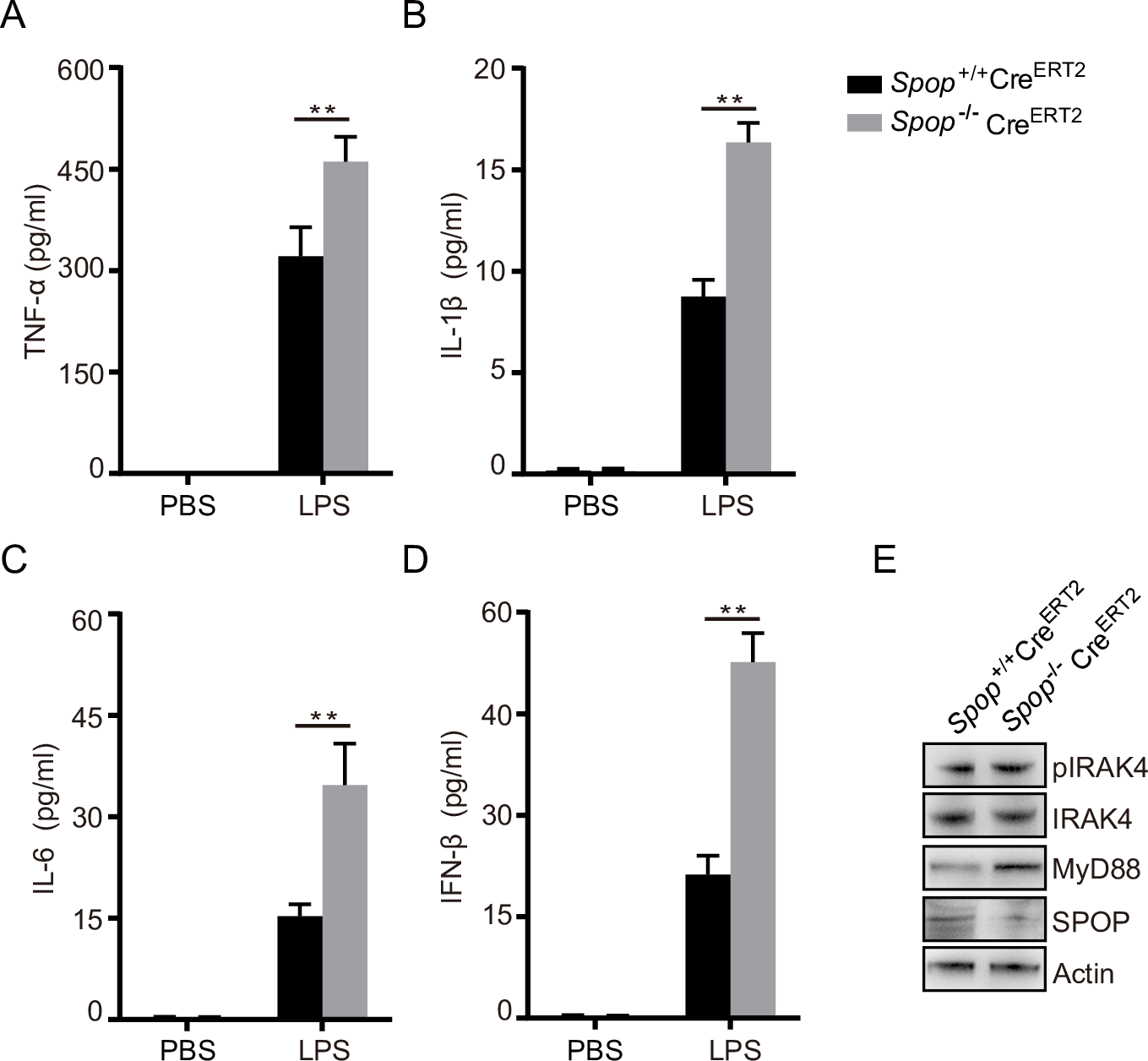

Supplement: S6 Fig — (A), (B), (C), and (D) ELISA of TNF-α, IL-1β, IL-6, and IFN-β in BMDMs supernatants from LPS-challenged Spop+/+CreERT2 and Spop-/-CreERT2 mice. **p < 0.01, error bars reflect ±SD. (E) Immunoblot analysis of BMDMs whole-cell lysates from Spop+/+CreERT2 and Spop-/-CreERT2 mice with indicated antibodies. (TIF) [file ppat.1008188.s006.tif]

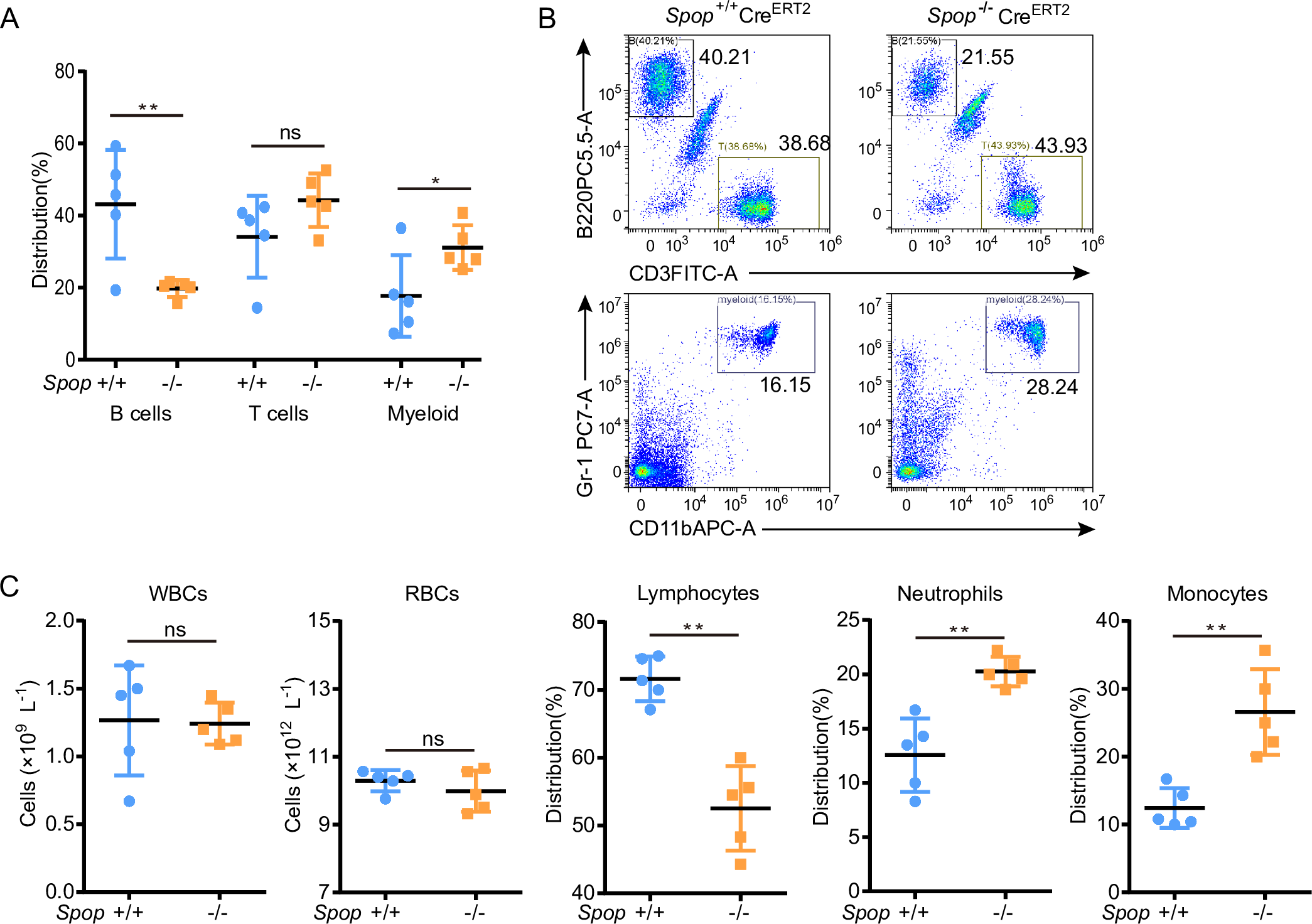

Supplement: S7 Fig — (A) Percentages of B (B220+), T (CD3+), and myeloid (CD11b+Gr-1+) cells in peripheral blood (n = 5). (B) Representative flow cytometry analysis plots of the proportions of B (B220+), T (CD3+), and myeloid (CD11b+Gr-1+) cells in peripheral blood (n = 5). (C) Counts of white and red blood cells and percentages of lymphocytes and neutrophils in peripheral blood of Salmonella-challenged Spop-/-CreERT2 and Spop+/+CreERT2 mice (n = 5). (TIF) [file ppat.1008188.s007.tif]
