# Supplementary material for: SPOP promotes ubiquitination and degradation of MyD88 to suppress the innate immune response
Source: PLoS Pathog. 2020 May 4;16(5):e1008188. doi: 10.1371/journal.ppat.1008188 (PMC7224567; doi:10.1371/journal.ppat.1008188)
Supplement: S1 Table — (DOCX) [file ppat.1008188.s008.docx]

**KEY RESOURCES TABLE**

| **REAGENT or RESOURCE** | **SOURCE** | **Catlog number** |
| --- | --- | --- |
| **Antibodies** | | |
| anti-FLAG | Abmart | M20008L |
| anti-GFP | Abmart | M20004L |
| anti-Myc | Abmart | M20002L |
| anti-SPOP | Santa Cruz | sc-377206 |
| anti-MyD88 | Cell Signaling Technology | 4283 |
| anti-IRAK4 | Cell Signaling Technology | 4363 |
| anti-Phospho-IRAK4 (Thr345/Ser346) | Cell Signaling Technology | 11927 |
| mAB to IgG | Abcam | ab131366 |
| Alexa Fluor 488 goat anti-mouse IgG(H+L chain) | Proteintech | SA00006-1 |
| Alexa Fluor 594 goat anti-mouse IgG(H+L chain) | Proteintech | SA00006-4 |
| Anti-Beta actin | Proteintech | 60008-I-Ig |
| Anti-HA | Abcam | Ab18181 |
| Anti-Myc (for immunofluorescence) | Cell Signaling Technology | 2272 |
| Protein A/G Agarose | Abmart | A10001M |
| Anti-Flag-tag mouse mAb(Agarose conjugated) | Abmart | M20018L |
| PE/Cy5 anti-mouse/human CD45R/B220 Antibody | Biolegend | 103209 |
| FITC anti-mouse CD3 Antibody | Biolegend | 100204 |
| PE/Cy7 anti-mouse Ly-6G/Ly-6C (Gr-1) Antibody | Biolegend | 108416 |
| APC anti-mouse/human CD11b Antibody | Biolegend | 101212 |
| **Reagents** | | |
| Lipopolysaccharides from Escherichia coli O55:B5 | Sigma | L4524 |
| Tamoxifen | Sigma | T5648 |
| MG132 | Abcam | ab141003 |
| DAPI | Beyotime | C1005 |
| Bafilomycin A1 | Cell Signaling Technology | 54645 |
| Bortezomib | MedChemExpress | HY-10227 |
| Cycloheximide | MedChemExpress | HY-12320 |
| Chicken IL-1B / IL-1 Beta ELISA Kit | LifeSpan BioSciences | LS-F4677 |
| Mouse IL-6 ELISA kit | Abcam | ab100712 |
| Mouse IL-1β ELISA kit | Abcam | ab100705 |
| Mouse IFN-β ELISA kit | Abcam | ab252363 |
| Mouse IFN-α ELISA kit | Abcam | ab252352 |
| Mouse TNF-α ELISA kit | Abcam | ab208348 |
| Lipofectamine® 3000 | Life technologies | L3000015 |
| TransIT-TKO® Transfection Reagent | Mirus | MIR2150 |
| Recombinant Murine M-CSF | PeproTech | 315-02 |
| Protease Inhibitor Cocktail | MedChem Express | HY-K0011 |
| Dual-Glo Luciferase Assay System | Promega | E2940 |
| Immobilon western chemilum HRP substrate | Merck | WBKLS0500 |
| **Oligonucleotides** | | |
| siRNA targeting sequence:SPOP  GCCAGAACACUAUGAACAUTT | This paper | N/A |
| **Recombinant DNA** |  |  |
| pcDNA3.1 | Thermo Fisher |  |
| pBI-CMV | Clontech | 631632 |
| pGL4.32[luc2P/NF-κB-RE/Hygro] Vector | Promega | E849A |
