# Supplementary material for: SPOP promotes ubiquitination and degradation of MyD88 to suppress the innate immune response
Source: PLoS Pathog. 2020 May 4;16(5):e1008188. doi: 10.1371/journal.ppat.1008188 (PMC7224567; doi:10.1371/journal.ppat.1008188)
Supplement: Attachment — Submitted filename: Response to Reviewers.docx [file ppat.1008188.s009.docx]

Reviewer's Responses to Questions
**Part I - Summary**
Please use this section to discuss strengths/weaknesses of study, novelty/significance, general execution and scholarship.

Reviewer #1: In the manuscript titled as “SPOP Promotes Ubiquitination and Degradation of MyD88 to Suppress the Innate Immune Response” by Dr. Zhao and the colleagues. Starting from the consensus sequences for SPOP-interacting domain, they went on to demonstrate that SPOP as an E3 ubiquitin ligase adaptor interacted with, and ubiquitylated MyD88, a key regulator in innate immune response, thus targeting MyD88 for degradation. On functional side, they clearly demonstrated that SPOP-mediated MyD88 ubiquitylation led to marked suppression of LPS-activated NF-kb signaling. Having mapped the sites for ubiquitylation in MyD88, they have nicely shown that ubiquitylation on some of the sites were specifically required for suppressing the activation of NF-kb pathway by LPS. Using siRNA and SPOP KO mice, the authors were able to show that endogenous SPOP was indeed a negative regulator of innate immune response, and SPOP deficient mice were susceptible to microbe infection, in this case, Salmonella typhimurium.
Overall, this work was well thought of and executed in a technically sound way. This work should be of interest by identifying SPOP as a new regulator of innate immune response.

Reviewer #2: In this paper, the authors identify an interaction between SPOP and MyD88 and attempt to demonstrate that SPOP promotes the degradation of MyD88 through K48-linked ubiquitination, and thus regulates its function. I understand that two other groups published on this very topic recently (Jin et al, Leukemia 2019; Guillamot et al, Nat Immunol 2019) and therefore these authors are eager to publish their work. Overall I am convinced that SPOP is in fact a regulator of MyD88 and am supportive of publication, but some major issues need to be addressed first.

Reviewer #3: This manuscript from Quinghe Li. et al., claims to describe a new role for the E3 ubiquitin ligase adaptor SPOP in controlling innate immune signaling by promoting MYD88 ubiquitination and subsequent degradation. However, two other papers have recently described very similar results and conclusions (Guillamot et al., Nature Immunology 2019 and Jin X et al., Leukemia 2019). Hence, it is very surprising that the authors are not aware of these publications and they do not make any reference to these studies. In addition to the lack of novelty, the conditional SPOP KO mouse model is poorly described, the in vivo experiments are not properly designed and therefore, the results are inconclusive. Taken together all these issues, I recommend that the authors strength their work before considering it for publication. **Part II – Major Issues: Key Experiments Required for Acceptance**
Please use this section to detail the key new experiments or modifications of existing experiments that should be absolutely required to validate study conclusions.
Generally, there should be no more than 3 such required experiments or major modifications for a "Major Revision" recommendation. If more than 3 experiments are necessary to validate the study conclusions, then you are encouraged to recommend "Reject".

Reviewer #1: However, when coming to the detailed dissection of the mechanism, several key experiments were missing that will be listed below, not necessarily in the order of relative importance:
1. When address the effect of POP-mediated MyD88 ubiquitylation on the stability of MyD88, a chase experiment using cycloheximide (CHX) should be performed to exclude the potential interference at the level of MyD88 transcription and/or its mRNA translation. Inhibitors like bortezomib and bafilomycin A specific for proteasome and autophagy pathway, respectively, should be tested on MyD88 stability in presence or absence of SPOP.
2. In the current version of the manuscript, it was shown that POP-mediated MyD88 ubiquitylation mainly resulted in poly-Ub chains in K48 linkages (Fig. 3A). Typically, this should be complemented with the experiment using K48R Ub mutant.

Reviewer #2: Figure 2B, 2D and 2E are not very convincing. For 2B: Perhaps the siRNA experiment should be repeated for improved knockdown of SPOP. For 2D-2E: It may help to use a commercially available antibody for endogenous MyD88 to see a greater effect on the overexpression of SPOP.
Much of Figure 3 is not convincing. Specifically, for the deletion analysis in Figure 3B-3C, this needs to be done using the same tag (ie Flag) on the same gel, with proper controls (i.e., WT Flag-MyD88). Also, what happens with Delta-INT in these experiments? I suggest Figure 3E simply be removed as it is not clear what is happening with these combination mutations, and to go straight into the point mutation analysis of Figure 3F.

Reviewer #3: Major points:
1. First of all, the authors should cite the previous work and clarify: 1) which experiments are replicates/similar to already publish work and which are novel findings, 2) how their results correlate with already published data.
2. Figure 3 describes the different lysines in chMYD88 which are ubiquitinated by SPOP. On this figure relies the novelty of this study as many of the other experiments/conclusions had already been shown. Therefore, I would recommend to complement and strength this data by corroborating similar observations in human MYD88. In addition, does over-expression MYD88 mutants induced increased pro-inflammatory cytokine release?
3. Figure 5 describes an in vivo function of SPOP in response to infections. However, the authors do not demonstrate that deletion of SPOP is efficient in their conditional model the and experiments are not properly design and the conclusions are unclear. Specifically:
a. The authors claimed that they generated a conditional KO mouse but they do not provide any description on how they made it. Which exons are targeted? Which specific Cre are they using?
b. In the methods the authors explain that they generate “Spop-/- mice by crossing Spop-/- with Cre transgene C57BL/6.” This is wrong and confusing. You couldn’t generate any conditional KO mice following this statement.
c. In the legend of Figure 5 the authors state: “Spop-/- heterozygous”. Again this is confusing as “-/-“indicates homozygosis
d. In the legend of Figure 5 the authors indicate that Spop was deleted by “feeding the mice with tamoxifen three times every 48 hours”. This is not the proper protocol to delete a gene with a Tamoxifen food and it raises concerns on the efficiency of the deletion.
e. They authors should provide western blot demonstrating that SPOP is efficiently deleted
f. To demonstrate that the phenotype is hematopoietic intrinsic, the authors should generate hematopoietic chimeras
g. The infection is done in only 4 mice and the differences in the survival curve relies in only one control. Therefore, authors should include more mice to ensure that the results are not biased by one outlier.
h. If deletion of SPOP results in MYD88 accumulation and subsequently, increased NF-kB response and pro-inflammatory cytokines release, one would expect an enhanced inflammatory response. Hence, it is unclear why the mice are more susceptible to infection rather than being able to clear more efficiently the pathogen.
i. Are pro-inflammatory cytokines increased in the serum of Spop KO mice? This is important because a sepsis shock could also explain the increase mortality of the mice.
j. Are the percentages of monocytes or neutrophils higher in the peripheral blood of the Spop KO mice than in the wild-type controls?
k. If the mice are more susceptible to infections, are Spop macrophages dysfunctional? How does it correlate with Myd88 dependent pathway?

**Part III – Minor Issues: Editorial and Data Presentation Modifications**
Please use this section for editorial suggestions as well as relatively minor modifications of existing data that would enhance clarity.

Reviewer #1: Minor issues:
Labels in many panels need to be more carefully tailored to help readers better understand the results. For example, in Fig.1D, for SPOP, the three major domains should be presented in FL, and aligned labels (those used in Fig.1E) of the full-length SPOP should be provided.
Language may need to be polished in many parts of the manuscript for accuracy and fluence.

Reviewer #2: Figure 4D: Is it possible that an insufficient knockdown is giving this very modest result? Can the authors repeat it and try to improve the knockdown efficiency?
Figure 5A: Not many mice were used in the two arms of the experiment (four each). What is the statistical analysis for this result (4/4 dying versus 3/4 dying)?
Abstract: Make it clear that these studies were done primarily in chicken (i.e., it is unclear to the reader what ‘chMyD88’ and ‘chSPOP’ are until later in the manuscript).
Introduction: Many other deubiquitinases besides OTUD4 regulate MyD88, and these should be discussed
The immunofluorescence analysis in Figure 1C is not useful and only reveals that the proteins are both present in the cytosol (even this is rather unclear). Either much higher resolution is necessary, or some other method should be used to demonstrate interaction (e.g., PLA), or this panel should just be removed.
It would be useful to label the different deletion mutants on the side of the panels with the names used in the adjacent panels in Figure 1 (i.e., “Delta-TIR” next to the “DD+INT”)

Some of the language used in the manuscript is slightly confusing and perhaps the manuscript could be improved by careful editing for language. For example, “To test whether the downregulation of SPOP on MyD88 is a common event among mammals” I believe the authors mean “To test whether the function of SPOP in downregulating MyD88 is conserved.”

Reviewer #3: 1. Authors should provide more information about the antibodies used in their experiments.
2. Do bone marrow macrophages from Spop KO mice show increased pro-inflammatory cytokines (TNF-a, il-1b, Il-6…) levels following LPS stimulation?
3. Quality of Figure 1C should be improved.
4. Figure 4C are very small and authors should include more numbers to ensure reproducibility.

Authors' response

Dear Editors

Thank you very much for your email of 19th Dec regarding the responses of the reviewers to our manuscript "SPOP Promotes Ubiquitination and Degradation of MyD88 to Suppress the Innate Immune Response" (PPATHOGENS-D-19-01994).

We appreciated all the responses and also thank the reviewers for their insightful comments concerning our work. We have considered their comments carefully and have answered all the points they raised. In addition, we have modified the author list to reflect the latest contributions made to the manuscript during revision. Besides the changes made according to the reviewers’ suggestion, we have simply removed the original S3 Fig showing the effect of CRISPRi of *spop* from the manuscript.

Our work was deeply delayed by the outbreak of coronavirus in China and we are not able to address one of the suggestions from reviewer 3 due to the epidemic. Currently the coronavirus is spreading all over the world and the situation is extremely serious in the United States, we sincerely hope that everything goes well with all of you.

All the best

Guiping Zhao, for the authors

**Part I - Summary**
Please use this section to discuss strengths/weaknesses of study, novelty/significance, general execution and scholarship.

Reviewer #1: In the manuscript titled as “SPOP Promotes Ubiquitination and Degradation of MyD88 to Suppress the Innate Immune Response” by Dr. Zhao and the colleagues. Starting from the consensus sequences for SPOP-interacting domain, they went on to demonstrate that SPOP as an E3 ubiquitin ligase adaptor interacted with, and ubiquitylated MyD88, a key regulator in innate immune response, thus targeting MyD88 for degradation. On functional side, they clearly demonstrated that SPOP-mediated MyD88 ubiquitylation led to marked suppression of LPS-activated NF-kb signaling. Having mapped the sites for ubiquitylation in MyD88, they have nicely shown that ubiquitylation on some of the sites were specifically required for suppressing the activation of NF-kb pathway by LPS. Using siRNA and SPOP KO mice, the authors were able to show that endogenous SPOP was indeed a negative regulator of innate immune response, and SPOP deficient mice were susceptible to microbe infection, in this case, Salmonella typhimurium.
Overall, this work was well thought of and executed in a technically sound way. This work should be of interest by identifying SPOP as a new regulator of innate immune response.
Response: We thank this referee for the support regarding our findings. We highly appreciate this referee’s valuable suggestions to improve our manuscript.

Reviewer #2: In this paper, the authors identify an interaction between SPOP and MyD88 and attempt to demonstrate that SPOP promotes the degradation of MyD88 through K48-linked ubiquitination, and thus regulates its function. I understand that two other groups published on this very topic recently (Jin et al, Leukemia 2019; Guillamot et al, Nat Immunol 2019) and therefore these authors are eager to publish their work. Overall I am convinced that SPOP is in fact a regulator of MyD88 and am supportive of publication, but some major issues need to be addressed first.
Response: Thank you. We highly appreciate your opinion and support of this manuscript.

Reviewer #3: This manuscript from Quinghe Li. et al., claims to describe a new role for the E3 ubiquitin ligase adaptor SPOP in controlling innate immune signaling by promoting MYD88 ubiquitination and subsequent degradation. However, two other papers have recently described very similar results and conclusions (Guillamot et al., Nature Immunology 2019 and Jin X et al., Leukemia 2019). Hence, it is very surprising that the authors are not aware of these publications and they do not make any reference to these studies. In addition to the lack of novelty, the conditional SPOP KO mouse model is poorly described, the in vivo experiments are not properly designed and therefore, the results are inconclusive. Taken together all these issues, I recommend that the authors strength their work before considering it for publication.

Response: We thank the reviewer so much for these constructive comments on our work. We really appreciate all the valuable suggestions raised by reviewer 3 and believe that they will help us improve our work dramatically. First of all, we would like to apologize to the reviewer for the incomplete information and mistakes in the manuscript. We have tried our best to address reviewer 3’s concerns by either clarifying certain aspects in the text, performing additional experiments, or citing evidence from similar publications.

We should have finished the revision before 17th Feb; however, many universities and institutes were shut down for almost two months because of the outbreak of a coronavirus in China, and we are now facing several dilemmas. Even now, only a few people have access to the lab, and that access is limited. We have addressed most of the suggestions of reviewer 3 that were critical for the main conclusions of our manuscript, but we are afraid that we would not be able to address the reviewer’s suggestion to map the ubiquitination sites of human MyD88 and investigate the function of human MyD88 mutants due to the great deal of work required to finish this, due to the unpredictable delay brought by the epidemic, and our eagerness to resubmit the revision as soon as possible since two similar works have been published. We sincerely hope that the reviewer understands our concerns.

**Part II – Major Issues: Key Experiments Required for Acceptance**

Reviewer #1: However, when coming to the detailed dissection of the mechanism, several key experiments were missing that will be listed below, not necessarily in the order of relative importance:
1. When address the effect of POP-mediated MyD88 ubiquitylation on the stability of MyD88, a chase experiment using cycloheximide (CHX) should be performed to exclude the potential interference at the level of MyD88 transcription and/or its mRNA translation. Inhibitors like bortezomib and bafilomycin A specific for proteasome and autophagy pathway, respectively, should be tested on MyD88 stability in presence or absence of SPOP.

Response: We thank the reviewer for this constructive suggestion to improve our manuscript. We have followed the reviewer’s suggestion concerning the CHX chase experiment. We knocked down the expression of *chSPOP* with RNAi and treated the cells with CHX for 0, 3, and 6 hours. The resulting Western blot showed that knockdown of *chSPOP* in the presence of CHX efficiently led to the accumulation of MyD88 protein, which meant that the accumulation of MyD88 was not due to bias in its transcription and translation. We have included this result as Fig 2B and modified the corresponding description in the manuscript.

Legend: Immunoblot analysis of endogenous chMyD88 in cells having *chSPOP* inhibited cells by siRNA. Cells were treated with 50 μg/ml CHX for 0, 3, or 6 hours.

In addition, we have treated the cells with bortezomib and bafilomycin A, which respectively inhibit the proteasome and the autophagy pathway. Like MG132, bortezomib treatment effectively rescued the degradation of MyD88 mediated by SPOP. In contrast, treatment with the autophagy inhibitor bafilomycin A had no effect on MyD88 degradation. These results further indicate that SPOP induces protein degradation through the proteasome pathway. These findings are depicted in Fig S2C, and we have revised the corresponding description in the results.

Legend: Immunoblot analysis of chMyD88 in cell lysates of chicken DF1 cells transfected with chSPOP and treated with DMSO, 100 nM bafilomycin A, or 50 nM bortezomib.

2. In the current version of the manuscript, it was shown that SPOP-mediated MyD88 ubiquitylation mainly resulted in poly-Ub chains in K48 linkages (Fig. 3A). Typically, this should be complemented with the experiment using K48R Ub mutant.

Response: We agree with the reviewer’s suggestion. We have carried out an immunoprecipitation experiment with cells transfected with K48 or K48R mutant vectors. As expected, SPOP efficiently promoted K48-linked ubiquitination of MyD88 but not K48R-linked ubiquitination. We have added this result as S3A Fig and modified the description in the results.

Legend: Immunoblot analysis of immunoprecipitated chMyD88 from chicken DF1 cells transfected with indicated expression plasmids.

Reviewer #2:

1. Figure 2B, 2D and 2E are not very convincing. For 2B: Perhaps the siRNA experiment should be repeated for improved knockdown of SPOP. For 2D-2E: It may help to use a commercially available antibody for endogenous MyD88 to see a greater effect on the overexpression of SPOP.

Response: We have followed this suggestion and repeated the siRNA experiment; furthermore, we have treated the cells with the translation inhibitor cycloheximide (CHX) to exclude the potential interference of *chMyD88* transcription or translation. Our results showed that the siRNA used in the current study could efficiently knock down the expression of *chSPOP* and thus lead to the accumulation of chMyD88 protein in the presence of CHX. Taken together, the results support that chSPOP could lead to the decrease in chMyD88 protein at the post-translation level. The associated figure is now new Fig 2B and we have modified the corresponding description.

Legend: Immunoblot analysis of endogenous chMyD88 in cells having *chSPOP* inhibited cells by siRNA. Cells were treated with 50 μg/ml CHX for 0, 3, or 6 hours.

For Fig 2D and 2E, we followed the reviewer’s suggestion and used commercial antibodies for human and mouse MyD88 to detect the effect of SPOP overexpression on endogenous MyD88 in human and mouse cells. As expected, exogenous SPOP effectively decreased the level of endogenous MyD88 protein in both human and mouse cells. We now include these data as new Fig 2D and 2E.

Legend: Immunoblot of human MyD88 in A549 cells and mouse MyD88 in mouse CHO cells transfected with Myc-SPOP.

2. Much of Figure 3 is not convincing. Specifically, for the deletion analysis in Figure 3B-3C, this needs to be done using the same tag (ie Flag) on the same gel, with proper controls (i.e., WT Flag-MyD88). Also, what happens with Delta-INT in these experiments? I suggest Figure 3E simply be removed as it is not clear what is happening with these combination mutations, and to go straight into the point mutation analysis of Figure 3F.

Response: We have followed this suggestion. We replaced Figure 3B and 3C with a new figure (now Fig 3B) showing DD, TIR, and INT domain truncated and FLAG-tagged MyD88 on the same gel. We found that SPOP could efficiently downregulate wild-type and the DD and TIR domain truncated MyD88, but had no effect on INT domain deleted Myd88, which is consistent with the results in our original manuscript. These results clearly indicated a requirement of the INT domain for the degradation of MyD88 by SPOP. We have changed the corresponding text in the manuscript in accordance with the new figure.

Legend: Immunoblot analysis of lysates from chicken DF1 cells transfected with indicated plasmids.

The reviewer mentioned that some panels of Figure 3 might cause confusion. Accordingly, we have simplified the original Fig 3C, 3D, and 3E and moved them to supplemental Fig 3. We did this instead of deleting them altogether because these would help communicate why we selected lysine 118, 119, and 124 for ubiquitination analysis. In addition, we also simplified the schematic map for Figure 3F (now S3C Fig).

Reviewer #3: Major points:
1. First of all, the authors should cite the previous work and clarify: 1) which experiments are replicates/similar to already publish work and which are novel findings, 2) how their results correlate with already published data.

Response: Our manuscript is a back-to-back work with the papers of Guillamot et al., published in *Nature Immunology* in Aug 2019, and Jin X et al., published in *Leukemia* in Nov 2019. The submission system of *PLOS Pathogens* shows that the initial date of our submission was 30th Oct. This submission system has a voluntary and optional link for preprinting in BioRxiv, and we posted our manuscript in BioRxiv (bioRxiv 831586) on 5th Nov, which was 24 days before the paper in *Leukemia* was published.

We apologize for not being aware of the paper published in *Nature Immunology*. We finished the writing of our manuscript before that paper was published, and did not keep closely following recent progress on SPOP. In fact, before the paper in *Nature Immunology* was published, we had submitted our manuscript to another journal, but it was rejected. We can provide the submission record if necessary.

We greatly regret for our ignorance of the paper of Guillamot et al. We definitely would want to cite and discuss such similar publications, and have referred to these articles in our revision. All three papers show similar observations concerning the regulation of SPOP by NF-kB signaling through MyD88, but the three papers also have some differences, especially between the work published in *Leukemia* and the other two. We and Guillamot et al. both show strong evidence that SPOP promotes K48-linked ubiquitination and degradation of MyD88; however, Jin X et al. show that SPOP promotes MyD88 ubiquitination but not degradation. In fact, Jin X et al. did not show any effect of MyD88 ubiquitination mediated by SPOP, neither by leading to its degradation nor through other ways.

In the article published in *Nature Immunol*, Guillamot et al. have presented a more systematic mechanism with a huge amount of work behind it. Guillamot et al. showed that SPOP restrained the inflammatory activation of hematopoietic stem cells (HSCs). In particular, they showed that hematopoietic-specific deletion of *Spop* promotes lethal neutrophilia, loss of SPOP resulted in systemic inflammation that proceeded in an unresolved manner, and the sustained response in those HSCs resulted in a lethal phenotype. Mechanistic studies revealed that SPOP restricted inflammation by ubiquitinating the innate signal transducer MyD88. Meanwhile, In the paper published in *Leukemia*, Jin et al. revealed that SPOP bound to and regulated the NF-kB pathway induced through MyD88 in a nondegradative way. They also looked into the dysfunction of mutated SPOP in lymphoid malignancies.

For our part, we focused on the degradation of MyD88 by SPOP through K48-linked ubiquitination in chicken. This was the same mechanism documented by Guillamot et al., but we presented our work on a relatively small scale and in a different manner. Firstly, we revealed that SPOP promoted MyD88 degradation through the proteasome pathway by rescuing that degradation with MG132. Secondly, we have systematically identified the ubiquitination sites of chicken MyD88 and investigated the function of ubiquitination site mutants. Thirdly, Guillamot et al. wondered in their discussion whether MYD88 protein stability was also controlled by SPOP in the terminally-differentiated innate immune subsets that are important for the resolution of inflammation outside of the marrow. We showed that through regulation of MyD88, SPOP enhances NF-κB activation and proinflammatory cytokine production in chicken macrophages. In addition, we observed that bone marrow-derived macrophages from *Spop* conditional knockout mice displayed enhanced immune response to LPS challenge. Taken together, these findings are beneficial complementary results to the work of Guillamot et al. Fourthly, we demonstrated the effect of *Spop* knockout on a specific pathogen, *Salmonella typhimurium*, which caused more death in *Spop* conditional knockout mice. Lastly, we carried out our work primarily in chicken, further indicating the conserved regulation of MyD88 by SPOP and confirming the findings of Guillamot et al. in mice.

We understand the reviewer’s comment about novelty, since similar results have been published. However, papers from different groups that have independently identified the same phenomenon in parallel will increase confidence in the results of that work and confirm its reproducibility. We have cited the above two related publications in our revision and discussed their findings.

2. Figure 3 describes the different lysines in chMYD88 which are ubiquitinated by SPOP. On this figure relies the novelty of this study as many of the other experiments/conclusions had already been shown. Therefore, I would recommend to complement and strength this data by corroborating similar observations in human MYD88. In addition, does over-expression MYD88 mutants induced increased pro-inflammatory cytokine release?

Response: We are grateful to the reviewer’s suggestion. We already had a triple-mutant chicken MyD88 plasmid on hand, and used it to examine the effect of overexpression of mutant chicken MyD88 on the release of pro-inflammatory factors. Chicken macrophage cells transfected with MyD88 and the triple mutant were challenged with LPS for six hours and the supernatant collected for ELISA assay. Our results showed that the mutant MyD88 led to greater release of IL-1b. We added these results as new S4D Fig.

Legend: ELISA of IL-1β in chicken HD11 macrophages transfected with triple mutant MyD88.

We appreciate the reviewer’s suggestions for repeating these experiments in human, and we believe that would be really beneficial in terms of making our work more interesting. However, our lab was shut down for nearly two months (since late January) because of the outbreak of coronavirus in China. Considering the severe competition in this topic and that two similar studies have been published, and especially that it would require a great deal of extra work and time to map the ubiquitination sites of human MyD88 and to decipher the functions of human MyD88 mutants, we are afraid we would not be able to complete that work in the current study. We have presented the ubiquitination sites in MyD88 and the function of its mutants using chicken as a model animal, and we respectfully argue that the absence of similar work on human MyD88 would not affect the main conclusion of this work. We sincerely hope that the reviewer understands our concerns.

3. Figure 5 describes an in vivo function of SPOP in response to infections. However, the authors do not demonstrate that deletion of SPOP is efficient in their conditional model the and experiments are not properly design and the conclusions are unclear. Specifically:
a. The authors claimed that they generated a conditional KO mouse but they do not provide any description on how they made it. Which exons are targeted? Which specific Cre are they using?

Response: We apologize for the missing information. We have added a schematic diagram of the *Spop* conditional knockout allele as S5 Fig. Exons 5 and 6 of *Spop* were floxed with two *loxP* sites by CRISPR/Cas9-mediated targeting. *Fstl1-CreERT2* knock-in mice were obtained from the National Resource Center for Mutant Mice at Nanjing University. The mice were injected with tamoxifen to induce gene knockout at a dose of 175 mg/kg body weight every two days, for 3 doses total. We have added a description in the method section.

b. In the methods the authors explain that they generate “Spop-/- mice by crossing Spop-/- with Cre transgene C57BL/6.” This is wrong and confusing. You couldn’t generate any conditional KO mice following this statement.

Response: We apologize for the incomplete description. In this study, we generated a conditional *Spop^F/F^Cre^ERT2+/+^* strain. We injected the mice intraperitoneally with tamoxifen to drive the expression of Cre. Briefly, *Spop^F/-^* mice were firstly crossed with *Cre^ERT2+/+^* mice to get *Spop^F/-^Cre^ERT2+/-^* heterozygotes, then the resulting *Spop^F/-CreERT2+/-^* mice were crossed with individuals having the same genotype to generate *Spop^F/F^Cre^ERT2+/+^*. We have added a description in the method section. Please find this information in S5 Fig.

c. In the legend of Figure 5 the authors state: “Spop-/- heterozygous”. Again this is confusing as “-/-“indicates homozygosis

Response: We thank the reviewer for pointing out this mistake. It should be “homozygous”, and we have corrected the mistake in the manuscript.

d. In the legend of Figure 5 the authors indicate that Spop was deleted by “feeding the mice with tamoxifen three times every 48 hours”. This is not the proper protocol to delete a gene with a Tamoxifen food and it raises concerns on the efficiency of the deletion.

Response: We are so sorry for the miswriting, we did not induce gene knockout by feeding the mice tamoxifen. Actually, we injected the mice intraperitoneally with tamoxifen at a dose of 175 mg/kg body weight every other day, for three times total.

e. They authors should provide western blot demonstrating that SPOP is efficiently deleted.

Response: Actually, we have examined the SPOP protein level in the spleens of control and knockout mice, but we forgot to put it in the manuscript. Our results showed that SPOP expression was efficiently eliminated in the spleens of tamoxifen-injected mice. We have added this result as S5C Fig.

f. To demonstrate that the phenotype is hematopoietic intrinsic, the authors should generate hematopoietic chimeras

Response: We appreciate this suggestion. A previous study has shown that SPOP deficiency in adult mice promotes unrestrained neutrophilia that correlates to rapid lethality (Guillamot et al., *Nature Immunology* 2019). In order to confirm that the pathology was hematopoietic cell intrinsic, Guillamot et al. transplanted lethally-irradiated hosts with control or *Spop* hematopoietic conditional knockout mouse bone marrow cells. After donor-derived hematopoiesis was established, a single poly(I:C) injection was sufficient to induce severe lethal neutrophilia in the mice reconstituted with bone marrow from conditional knockout mice, confirming that the pathology was exclusively driven by *Spop* deletion in donor-derived cells. We also observed neutrophilia in *Spop* knockout mice in our study (new S7 Fig).

Furthermore, Guillamot et al. showed that poly(I:C), LPS, and influenza virus all led to neutrophilia and increased lethality in *Spop* conditional knockout mice. IL-1β stimulation functions as an emergency signal that directly instructs HSCs through activation of the AP-1 and NF-κB pathways and through the engagement of a PU.1-dependent myeloid gene program (Pietras, E. M. et al., Nat. Cell Biol., 2016). Guillamot et al. revealed that poly(I:C) induces a transient increase of IL-1β in the bone marrow, and this induction led to the development of the poly(I:C)-dependent phenotypes reported in hematopoietic-specific *Spop* deleted mice, such as neutrophilia. Like poly(I:C), LPS treatment would also trigger IL-1R signaling and an increase of IL-1β (Rubartelli et al., EMBO J, 1990; Fettelschoss et al., PNAS, 2011; Zaslona et al, J Immunol, 2017). Our results also demonstrated a significant increase of serum IL-1β in mice challenged by *Salmonella typhimurium* (Fig 5C-5E), and we made a similar observation of neutrophilia in *Salmonella typhimurium-*challenged *Spop*-deficient mice (S7 Fig). Although Guillamot et al challenged their hematopoietic chimeras only with poly(I:C), based on the common activation of IL-1R signaling, it is reasonable to speculate that *Salmonella typhimurium* would work in the same manner as it triggers an immune response through LPS. The results supporting this speculation, adapted from Guillamot et al (Nat Immunol, 20(9):1196-1207, 2019) are as follows:

Legend (Adopted from Guillamot et al. Nat Immunol, 2019): A, Kaplan–Meier analysis of survival of the indicated mice after a single poly(I:C) injection (n = 5 per genotype except for Spop^Δ^MxCre, which is n = 3; statistical analysis: Mantel–Cox test). B, Kaplan–Meier analysis of survival of the indicated hematopoietic chimeras after a sublethal LPS injection (n = 4). C, Kaplan–Meier analysis of survival of the indicated mice after intranasal influenza inoculation (n = 10). D, Percentage of myeloid (CD11b+Gr-1+) cells in the peripheral blood of Spop KO and control hematopoietic chimeras on d15 after poly(I:C) or PBS injection (n = 5 except for Spop^Δ/Δ^MxCre, which is n = 3). E, Percentage of myeloid (CD11b+Ly6G+) cells in the peripheral blood of the indicated mice on d15 after a sublethal LPS injection (n = 4). F, Percentage of myeloid (CD11b+Gr-1+) cells in peripheral blood of Spop KO Cre^ERT2^ (n = 5) and control (n = 4) hematopoietic chimeras at the indicated days after intranasal influenza inoculation.

Legend (Adopted from Guillamot et al. Nat Immunol, 2019): Kaplan-Meier analysis of survival of Spop^+/+^MxCre and Spop^△/△^MxCre hematopoietic chimeras after donor hematopoietic reconstitution and one injection of Poly(I:C) (n=5 wild-type, n=6 Spop KO. Statistical analysis: Mantel-Cox test).

We thank the reviewer for the suggestion to generate hematopoietic chimeras to show that the phenotype we observed is hematopoietic intrinsic, and accept that it should be included for deepening understanding of the mechanism. However, our work focused on the regulation of MyD88 by SPOP, and our purpose in generating a mouse model was to examine disease susceptibility. We respectfully argue that the request to address if the phenotype is hematopoietic intrinsic could be efficiently explained by the above findings, and have added the relevant information to the discussion to make the mechanism more clear to the reader.

g. The infection is done in only 4 mice and the differences in the survival curve relies in only one control. Therefore, authors should include more mice to ensure that the results are not biased by one outlier.

Response: We thank the reviewer for pointing this out. We have challenged *Spop* conditional knockout mice again and extended the number of mice to seven in both control and knockout groups. We found that the *Spop* conditional knockout mice were more susceptible to *Salmonella* infection. We have updated Fig 5A to reflect this result.

Legend: Mice were challenged with *Salmonella typhimurium* and their survival rates monitored for 36 hours (n = 7 each group; statistical analysis: Mantel–Cox test).

h. If deletion of SPOP results in MYD88 accumulation and subsequently, increased NF-kB response and pro-inflammatory cytokines release, one would expect an enhanced inflammatory response. Hence, it is unclear why the mice are more susceptible to infection rather than being able to clear more efficiently the pathogen.

Response: Uncontrolled innate immune response results in overwhelming response to an infection. Previous studies have shown that knockdown of genes regulating the innate immune pathway causes increased innate immune response, but also more susceptibility to infections (Bachmaier et al., Nature Med, 2007; Han et al., Nature Immunol, 2010; Galbas et al., J Immunol, 2017). For example, genetic deletion of the E3 ubiquitin ligase *Cblb* in mice promotes the TLR4-mediated acute inflammatory response (Bachmaier et al., Nature Med, 2007). Loss of *Cblb* significantly increases sepsis-induced release of inflammatory cytokines and chemokines, leading to higher mortality. Han et al. have shown that Integrin CD11b (encoded by *Itgam*) negatively regulates TLR-triggered inflammatory responses by activating Syk and promoting degradation of MyD88 (Han et al., Nature Immunol, 2010). *Itgam* knockout mice are more susceptible to *E. coli* infection and produced more of the tumor necrosis factor IL-6 than did heterozygous control mice. Deletion of *Spop* would lead to aberrant accumulation of MyD88 and thus increased NF-kB pathway activity, which ultimately leads to more release of pro-inflammatory cytokines. In our study, we speculate that the conditional knockdown of *Spop* in mice resulted in aberrant release of inflammatory cytokines, and thus the KO mice are more susceptible to *Salmonella* infection. In response to this question, we show evidence of significantly increased levels of pro-inflammatory cytokines in the KO mice (new Fig 5C-5E).

i. Are pro-inflammatory cytokines increased in the serum of Spop KO mice? This is important because a sepsis shock could also explain the increase mortality of the mice.

Response: We appreciate this suggestion. We measured the concentrations of proinflammatory cytokines in the sera of *Salmonella*-challenged mice. We found that the concentrations of pro-inflammatory cytokines like IL-1β, IL-6 and IFN-α were significantly greater in serum from *Spop* conditional knockout mice than in serum from wild-type controls. These results are consistent with the finding that negative regulation of the TLR4-MyD88-mediated acute inflammatory response results in more susceptibility to infections (Bachmaier et al., Nature Med, 2007; Han et al., Nature Immunol, 2010). We have added these results to the revised manuscript as new Fig 5C-5E.

j. Are the percentages of monocytes or neutrophils higher in the peripheral blood of the Spop KO mice than in the wild-type controls?

Response: We have followed this suggestion and analyzed the percentages of monocytes, neutrophils, white blood cells, and lymphocytes in the peripheral blood of mice six hours after *Salmonella* infection. Consistent with the results described by Guillamot et al., we observed significant increase in the percentage of neutrophils and platelets in *Spop* conditional knockout mice compared with wild-type controls, along with significant decreases in the percentage of lymphocytes. Our results indicate that *Spop*-deficient mice might develop neutrophilia that correlates with higher susceptibility to acute *Salmonella* infection. We have added these results as S7 Fig.

Legend: Hematological characteristics of *Spop* conditional knockout mice. (A) Percentages of B (B220^+^), T (CD3^+^), and myeloid (CD11b^+^Gr-1^+^) cells in peripheral blood (n = 5). (B) Representative flow cytometry analysis plots of the proportions of B (B220^+^), T (CD3^+^), and myeloid (CD11b^+^Gr-1^+^) cells in peripheral blood (n = 5). (C) Counts of white and red blood cells and percentages of lymphocytes and neutrophils in peripheral blood of *Salmonella*-challenged *Spop*^-/-^Cre^ERT2^ and *Spop*^+/+^Cre^ERT2^ mice (n = 5).

k. If the mice are more susceptible to infections, are Spop macrophages dysfunctional? How does it correlate with Myd88 dependent pathway?

Response: Thanks for these constructive questions. We have isolated bone marrow cells, induced them to differentiate into macrophages, and challenged the cells with LPS for six hours. Cell supernatants were collected for ELISA analysis. Our results demonstrated that macrophages from *Spop*-deficient mice produced more pro-inflammatory factors than those from controls (S6A-S6D Fig).

We next investigated if the dysfunction was associated with MyD88-dependent pathway. We observed dramatic MyD88 accumulation and Irak4 hyper-phosphorylation in *Spop* conditional knockout mice (S6E Fig).

**Part III – Minor Issues: Editorial and Data Presentation Modifications**
Reviewer #1: Minor issues:
1. Labels in many panels need to be more carefully tailored to help readers better understand the results. For example, in Fig.1D, for SPOP, the three major domains should be presented in FL, and aligned labels (those used in Fig.1E) of the full-length SPOP should be provided.

Response: We thank the reviewer for pointing this out. We have revised Fig 1D and 1F and labeled the truncations with names on the right of the panels. We have also revised the schematic map of MyD88 in Fig 3.

Legend: (Left) Schematic presentation of chMyD88 and its truncation mutants. FL, full-length; DD, death domain; INT, intermediate domain. TIR, Toll Toll/interleukin-1 receptor homology domain. (Right) Schematic diagram of chSPOP and its truncation mutants. MATH, meprin and TRAF homology domain; BTB, bric-a-brac, tramtrack and broad complex/POZ domain; BOX, 3-box domain together with the C-terminal nuclear localization sequence.

2. Language may need to be polished in many parts of the manuscript for accuracy and fluence.
Response: We followed the reviewer’s suggestion. The whole paper has been re-edited by a professional native English speaker.

Reviewer #2:

1. Figure 4D: Is it possible that an insufficient knockdown is giving this very modest result? Can the authors repeat it and try to improve the knockdown efficiency?

Response: Thanks for your suggestion. We have rerun the experiment and recreated Figure 4D.

2. Figure 5A: Not many mice were used in the two arms of the experiment (four each). What is the statistical analysis for this result (4/4 dying versus 3/4 dying)?

Response: For statistical analysis, we used the Mantel-Cox test. In order to confirm the reproducibility of our *Salmonella* infection results, we have included more mice (n=7 for each group), and the data supported that *Spop* knockout mice were more susceptible to *Salmonella* infection (Fig 5A).

Legend: Mice were challenged with *Salmonella typhimurium* and their survival rates monitored for 36 hours (n = 7 each group; statistical analysis: Mantel–Cox test).

3. Abstract: Make it clear that these studies were done primarily in chicken (i.e., it is unclear to the reader what ‘chMyD88’ and ‘chSPOP’ are until later in the manuscript).

Response: As the reviewer suggested, we have revised the abstract to make it clear that *chMyD88* and *chSPOP* were abbreviations for chicken genes.

4. Introduction: Many other deubiquitinases besides OTUD4 regulate MyD88, and these should be discussed

Response: We appreciate this suggestion. Two protein deubiquitinases could deubiquitinate MyD88: OTUD4 and CYLD. We have discussed these two deubiquitinases in the manuscript.

5. The immunofluorescence analysis in Figure 1C is not useful and only reveals that the proteins are both present in the cytosol (even this is rather unclear). Either much higher resolution is necessary, or some other method should be used to demonstrate interaction (e.g., PLA), or this panel should just be removed.

Response: Thank you for your suggestions. We have redone the immunofluorescence analysis with a confocal microscope and replaced the figure.

6. It would be useful to label the different deletion mutants on the side of the panels with the names used in the adjacent panels in Figure 1 (i.e., “Delta-TIR” next to the “DD+INT”)
Response: We thank the reviewer for pointing this out. We have revised Fig 1D and 1F and labeled the truncations with names on the right of the panels. We have also revised the schematic map of MyD88 in Fig 3.

Legend: (Left) Schematic presentation of chMyD88 and its truncation mutants. FL, full-length; DD, death domain; INT, intermediate domain. TIR, Toll Toll/interleukin-1 receptor homology domain. (Right) Schematic diagram of chSPOP and its truncation mutants. MATH, meprin and TRAF homology domain; BTB, bric-a-brac, tramtrack and broad complex/POZ domain; BOX, 3-box domain together with the C-terminal nuclear localization sequence.

7. Some of the language used in the manuscript is slightly confusing and perhaps the manuscript could be improved by careful editing for language. For example, “To test whether the downregulation of SPOP on MyD88 is a common event among mammals” I believe the authors mean “To test whether the function of SPOP in downregulating MyD88 is conserved.”
Response: We thank the reviewer for this suggestion. We have revised the corresponding sentence and the whole paper has been re-edited by a professional native English speaker.

Reviewer #3:

1. Authors should provide more information about the antibodies used in their experiments.

Response: we have listed all the antibodies and reagents used in this study as supporting table 1.
2. Do bone marrow macrophages from Spop KO mice show increased pro-inflammatory cytokines (TNF-a, il-1b, Il-6…) levels following LPS stimulation?

Response: We have isolated bone marrow cells, induced them to differentiate into macrophages, and challenged the cells with LPS for six hours. Cell supernatants were collected for ELISA analysis. Our results demonstrated that macrophages from *Spop*-deficient mice produced more proinflammatory factors than did the controls (S6 Fig).

3. Quality of Figure 1C should be improved.

Response: Thank you for your suggestion. We have redone the immunofluorescence analysis with a confocal microscope and replaced the figure.

4. Figure 4C are very small and authors should include more numbers to ensure reproducibility.

Response: We believed the reviewer meant Fig.4D. We have rerun the experiment and updated the figure.
